# Supplementary figures and images for: Influence of Dispersed TiO2 Nanoparticles via Steric Interaction on the Antifouling Performance of PVDF/TiO2 Composite Membranes
Source: Membranes (Basel). 2022 Nov 9;12(11):1118. doi: 10.3390/membranes12111118 (PMC9694972; doi:10.3390/membranes12111118)

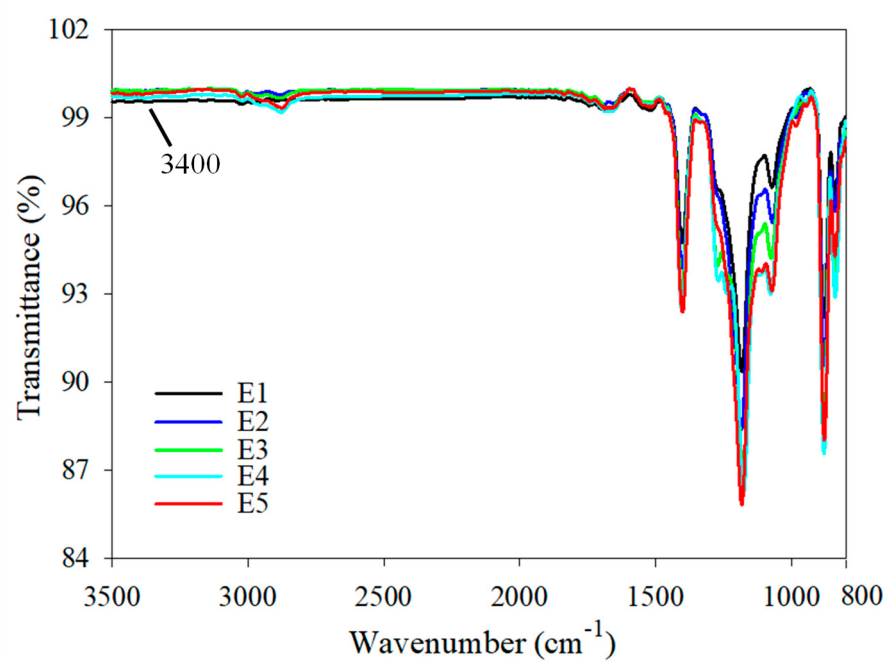

**Figure S1.** Full spectra of PVDF/TiO<sub>2</sub> composite membranes E1–E5 determined by ATR-FTIR.

Supplement: Supplementary file 1 [file membranes-12-01118-s001.zip › membranes-2026387-supplementary.pdf]
